# Spectral fusion-based breathing frequency estimation; experiment on activities of daily living

**DOI:** 10.1186/s12938-018-0533-1

**Published:** 2018-07-27

**Authors:** Iman Alikhani, Kai Noponen, Arto Hautala, Rahel Ammann, Tapio Seppänen

**Affiliations:** 1 Physiological Signal Analysis Team, Center for Machine Vision and Signal Analysis, University of Oulu, Pentti Kaiteran Katu 1, 90014 Oulu, Finland; 2 Swiss Federal Institute of Sport, Hauptstrasse 247, 2532 Magglingen, Switzerland

**Keywords:** Heart rate variability, Time–frequency analysis, Single-channel ECG, Breathing rate estimation, ECG morphology

## Abstract

**Background:**

We study the estimation of breathing frequency (BF) derived from wearable single-channel ECG signal in the context of mobile daily life activities. Although respiration effects on heart rate variability and ECG morphology have been well established, studies on ECG-derived respiration in daily living settings are scarce; possibly due to considerable amount of disturbances in such data. Yet, unobtrusive BF estimation during everyday activities can provide vital information for both disease management and athletic performance optimization.

**Method and data:**

For robust ECG-derived BF estimation, we combine the respiratory information derived from R–R interval (RRI) variability and morphological scale variation of QRS complexes (MSV), acquired from ECG signals. Two different fusion techniques are applied on MSV and RRI signals: cross-power spectral density (CPSD) estimation and power spectrum multiplication (PSM). The algorithms were tested on large sets of data collected from 67 participants during office, household and sport activities, simulating daily living activities. We use spirometer reference BF to evaluate and compare our estimations made by different models.

**Results and conclusion:**

PSM acquires the least average error of BF estimation, $\%D^{2\sigma }=9.86$ and $\%E = 9.45$, compared to the reference spirometer values. PSM offers approximately 25 and 75% less error in comparison with the CPSD fusion estimation and the estimation by those two exclusive sources, respectively. Our results demonstrate the superiority of both of the fusion approaches, compared to the estimation derived from either of RRI or MSV signals exclusively.

## Background

Breathing frequency (BF) is a vital biomarker utilized for diagnostics, and sport physiology applications. However, measuring BF using respiratory sensors over long-term monitoring sessions can be uncomfortable. Indirect monitoring of respiratory frequency can be conducted in different modalities, including video-based [1], electrical impedance pneumography-based [2] or wearable accelerometer-based respiration reconstruction [3]. Single or multiple channel electrocardiography (ECG) is one of the well-established modalities explored for BF and respiratory pattern reconstruction, known as ECG-derived respiration (EDR).

EDR was introduced during the 80s in [4, 5]. The idea was based on the fact that respiratory sinus arrhythmia (RSA)—which is obtainable from heart rate variability (HRV)—correlates with the respiratory pattern. Additionally, the beat-to-beat morphological variation of ECG signal, e.g. modulation of R-peak amplitudes or areas under R-wave, is largely a result of inhale and exhale. Thus, it is feasible to derive respiratory information such as the BF indirectly by analyzing a single-channel ECG signal.

The development of wearable devices has made it practical and inexpensive to monitor the biosignals of subjects who can benefit from continuous monitoring, including patients suffering from sleep apnea [6–8], and professional athletes managing their exercise regimes according to the biosignal feedback [9]. Consequently, there is an increasing demand on the biosignal processing algorithm development to enhance the capabilities of wearables and/or to introduce new features.

In order to rely on BF reconstruction from single-channel wearable ECG signal, the algorithm employed and its performance should be validated across a variety of daily activities. We found a few studies about BF estimation during physical activities using single-channel ECG signals. However, most of the research in this discipline has contributed to the resting state EDR [10–12]. What is more, severe challenges are introduced to ECG processing in physical activity contexts, including variable mean heart rate (HR), high level of movement artifacts and introduction of cardio–locomotion coupling (CLC) components [13, 14].

In this paper, we hypothesize that the combined information taken from different existing sources of respiratory signal in ECG indices yields a more accurate and reliable BF estimation. To this end, we tested two frequency-domain data fusion techniques on two respiratorily modulated indices. Namely, we apply cross-power spectral density (CPSD) estimation and power spectrum multiplication (PSM) on R–R interval variability (RRI) and morphological scale variation of QRS complexes (MSV). We tested our hypothesis on a data set collected from 67 subjects in real-life activities such as office, households and sports.

## Methods

### Preprocessing

**Fig. 1:**
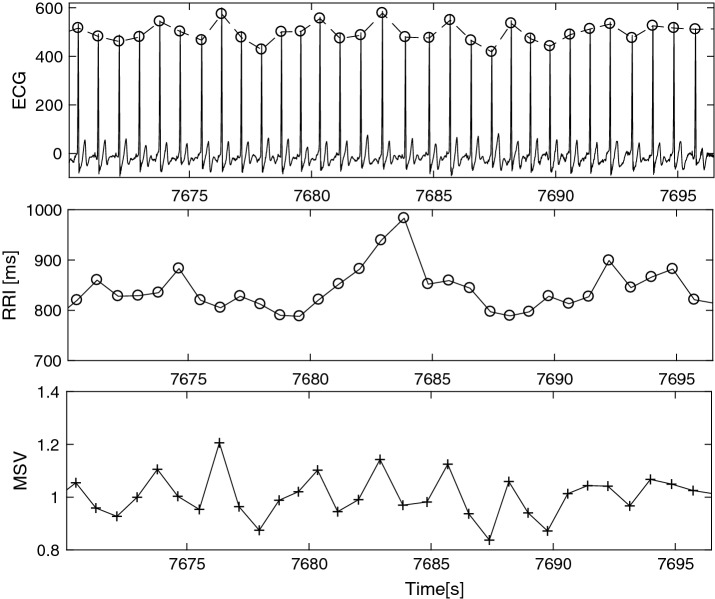
Derived signals from ECG, potentially containing respiratory frequency information. The first row is a 30-s ECG signal and in the next rows corresponding RRI and MSV signals are depicted, respectively

The first step is to preprocess the raw ECG and extract the RRI and MSV indices. Initially, we detect the R-peak locations using the conventional Pan-Tompkins method [15]. Then, RRI is simply obtained by computing the intra-beat intervals and MSV is constructed by the procedure introduced in [16], following the R-peak delineation. MSV at each candidate R-time instant is the morphological shape difference of the candidate QRS complex with the mean shape of QRS-complexes. According to the literature, these signals potentially contain respiratory components within their spectrum [4, 16]. Figure 1 exemplifies the constructed signals as well as the original ECG for a short time window.

This preprocessing step is followed by signal conditioning wherein anomalies from RRI are detected and replaced with linear interpolation to keep the number of beats unaltered. This ectopic beat detection and editing is explained in more details in the following subsection.

### Ectopic beat detection and editing

In practice, the RRI signal derivation must be followed by ectopic and anomaly beat detection and editing [17, 18]. Especially when the data is recorded during physical activities via wearables, the signal quality of ECG is generally lower, which can cause problems in the R-peak detection. During such activities, motion artifacts and ectopic beats are also more abundant. Hence, ectopic beat detection and editing is an important preprocessing step in HRV analysis.

Let’s assume that RRI signal consists of *n* samples, and every sample can be expressed as $RR_i.$ The following steps illustrate the proposed procedure of anomaly detection and editing:

- Detect the evident outliers and edit them using linear interpolation. Although the HR ranges from 60 beats/min (bpm) to 200(bpm) in our dataset, we define a wider range for healthy intervals between 250 and 1500 ms. This is to ensure that healthy controlled beats are not discarded and to make the outlier detector more general for any dataset.
- Construct an n-by-three dimensional feature matrix describing local HRV around each beat. More precisely, the following three descriptors are calculated for each beat:
$$RR_i,$$
$$\mid RR_i-RR_{i-1}\mid +\mid RR_i-RR_{i-2}\mid$$
$$\mid RR_i-RR_{i+1}\mid +\mid RR_i-RR_{i+2}\mid$$
  At the borders, use symmetric padding by mirroring the samples.
  ($$\{RR_1, RR_2, RR_3,\ldots , RR_{n-2}, RR_{n-1}, RR_n\} \Longrightarrow$$
  $$\{RR_3, RR_2, RR_1, RR_2, RR_3,\ldots , RR_{n-2}, RR_{n-1}, RR_n, RR_{n-1}, RR_{n-2}\}$$).
  Duration unit of millisecond is used.
- Normalize the feature matrix column-wise for zero-mean and unit-standard deviation.
- A multivariate Gaussian distribution is fitted on the feature matrix using $\mu \in \mathrm{I\!R}^3$ and the covariance matrix of features $\sigma \in \mathrm{I\!R}^{3\times 3}$. Given the feature set $\{ x^{(1)}, x^{(2)},\ldots , x^{(n)} \}$, where every $x^{(i)} \in \mathrm{I\!R}^3$,
  1 $$\begin{aligned} p(x;\mu ,\sigma ) = \frac{1}{(2\pi )^{\frac{n}{2}}\mid \sigma \mid ^{\frac{n}{2}}}e^{-\frac{1}{2}(x-\mu )^T\sigma ^{-1}(x-\mu )}. \end{aligned}$$
  The model can be fitted by computing
  2 $$\begin{aligned} \mu = \frac{1}{n}\sum ^n_{i=1}x^{(i)} \end{aligned}$$
  and
  3 $$\begin{aligned} \sigma =\frac{1}{n}\sum ^n_{i=1}(x^{(i)}-\mu )(x^{(i)}-\mu )^T \end{aligned}$$
- Detect as anomalous beats those with $p(x^{(i)})\le \epsilon$ where $\epsilon$ is a threshold value. The value of $\epsilon$ tunes the strictness of the anomaly detection and is adjusted according to the HR level. In this work $\epsilon$ is set in the range of $[10^{-11},10^{-5}]$, where lower range was used for high exercise intensity data, and upper range for non-physical context.
- Edit the detected anomalies using linear interpolation by nearby controlled beats to keep the same number of beats. Linear interpolation is selected because it is a widely used method.
- Steps (b) to (f) are iterated as long as there is a newly detected anomaly, otherwise the procedure is terminated.

After a few iterations—the number of which depending on the quality of the signal, number of ectopic beats and the value of $\epsilon$—the RRI signal is prepared for further steps. All the ectopic beats are replaced with the interpolated values in the MSV signal as well. Interpolation is made using a linear model over the MSV controlled beats.

### Spectral analysis

Naturally, the heart is beating irregularly. Hence, the constructed signals (RRI and MSV) are not evenly sampled. Therefore, we resample the signals to make them equidistant which also prepares them for spectral analysis, as suggested in [19]. We interpolate the signals by 8 Hz sampling frequency and compute their baseline using a 5-s window moving average smoothing function. We apply the standardization procedure in [20] for correction of mean HR, induced by physical activities as follows: The baseline is subtracted from the signal and divided up by the baseline, i.e. (signal-baseline)/baseline.

The HRV spectral components during physical activities differs from those of resting condition [14]. During steady state resting condition, high frequency (HF) components of HRV spans the range of [0.15 Hz 0.4 Hz] [21], but during exercise this range is redefined to be [0.15 Hz $\frac{HR_m}{2}],$ in which $\frac{HR_m}{2}$ is the half of local mean HR^1^ in Hertz.

Considering the heart as a natural signal sampler with the sampling frequency of $HR_m,$ $\frac{HR_m}{2}$ is the intrinsic Nyquist frequency of HRV signal, meaning that interpretable physiological components should fall within the range of HF. Aliasing phenomenon of the components over this intrinsic Nyquist frequency of HRV signal might lead to misinterpretation of autonomic nervous system (ANS) activity. For instance, let’s assume an abnormal BF just a bit over the intrinsic Nyquist frequency, e.g. $\frac{HR_m}{2}+\Delta F$. Due to the sampling theorem, it will fold back into the HF range of interest at the frequency of $\frac{HR_m}{2}-\Delta F$. In the spectral analysis this could lead into misguided detection of BF at this lower frequency, instead of the higher actual one. Speaking of which, a prevalent factor that might influence the spectral interpretation of HRV signal is CLC components that arise from cadence during walking or running; or pedaling frequency during cycling [13, 14]. Because of the mentioned aliasing phenomenon, these components will fold back to the HF range when they exceed the Nyquist frequency. The folded components might possess significant energy level compared to the energy at BF at some time instants. Our proposed fusion models aim to highlight the joint BF components in both signals and attenuate the influence of existing disturbances exclusively in either of those signals.

**Fig. 2:**
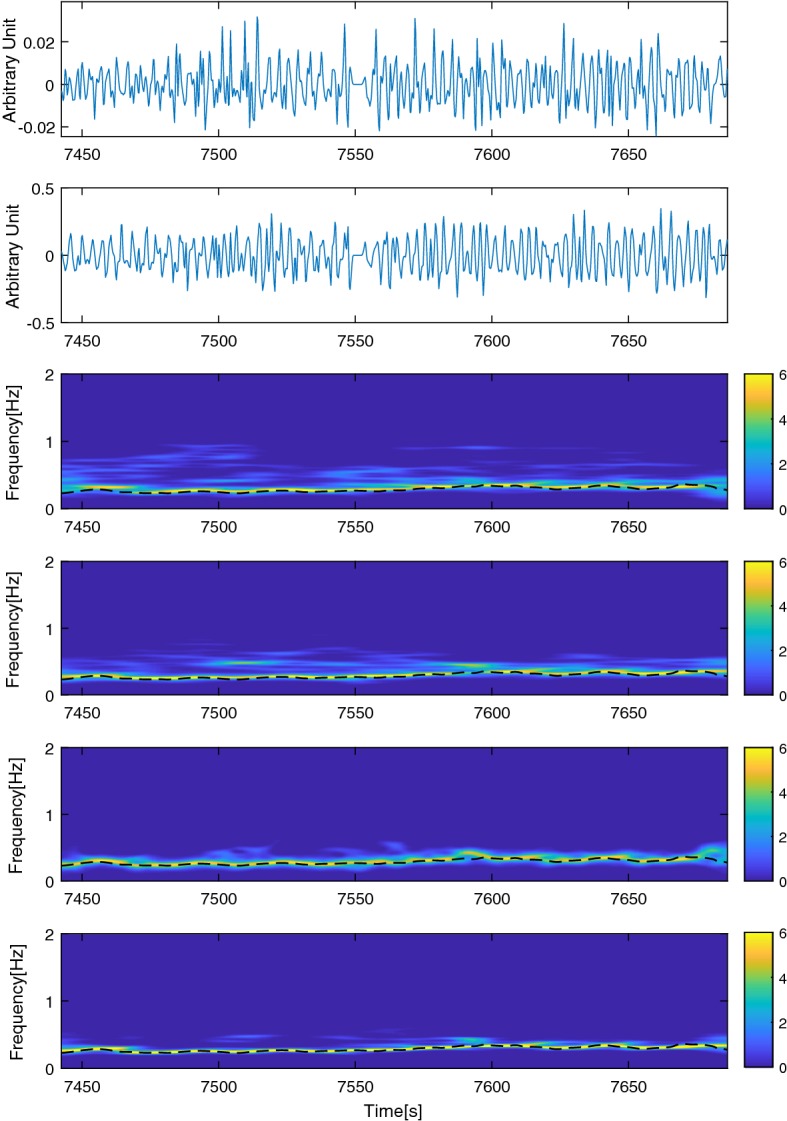
Preprocessed signals plus the representation of them and their fusions. This figure shows an epoch of the time series, RRI and MSV after preprocessing in the first and second top sub-figures. Assuming RRI and MSV signals as *x* and *y*, time–frequency representations of *x* and *y* are depicted in the third and fourth sub-figures ($\mathcal {N}_{Z-score}\{S_{xx}\}$ and $\mathcal {N}_{Z-score}\{S_{yy}\}$, respectively). Normalized squared magnitude of CPSD ($\mathcal {N}_{Z-score}\{\mid S_{xy}\mid ^2\}$) is represented in the second last sub-figure. The last spectrogram is the normalized spectral multiplication of MSV and RRI ($\mathcal {N}_{Z-score}\{\mid S_{xx} \cdot S_{yy}\mid \}$ ). $\mathcal {N}_{Z-score}\{.\}$ states as *Z*-score column-wise normalization. The dashed lines over the time–frequency representations are the reference BF

For spectral analysis, smoothed pseudo Wigner–Ville distribution (SPWVD) is preferred as the time–frequency representation model in this study. It is a nonparametric quadratic model and offers high time and frequency resolution [22]. According to [22], we define the SPWVD of *x*(*n*) as Eq. (4)

4 $$\begin{aligned} X(n,m)=2\sum ^ {N-1} _ {k=-N+1} \mid f(k)^2 \mid \sum ^ {M-1} _ {p=-M+1} t(p)r_x(n+p,k)e^{-\frac{2\pi jkm}{N}} \end{aligned}$$

where *x*(*n*) is a discrete signal, $r_x(n,k)$ is instantaneous autocorrelation function, *n* and *m* are the time and frequency indices. *f*(*k*) and *t*(*p*) are the frequency and time smoothing normed window, sized $2N-1$ and $2M-1$, respectively. The first four top sub-figures of Fig. 2 shows the two time series (RRI and MSV) as well as their normalized SPWVD time–frequency representations.

### Spectral fusion

The existence of energy around the BF range in HRV and morphologically-derived signals is justified in the literature as noted in the Introduction. Moreover, some of the possible challenges related to these signals were also described in the previous subsection.

To enhance the estimation of BF particularly during a non-stationary recording situation (e.g. physical activities), we propose to combine the spectral information of BF components of RSA and morphological variation (situated in RRI and MSV signals, respectively). We address the spectral fusion in two different ways: by PSM and CPSD estimation of MSV and RRI signals. The key point is that potentially there are mutual joint energies in the spectrum of these two signals, supposedly greater than the background energy and corresponding to the BF at each time instant. Thus, attempting to find the significant joint energy content present in both signals at each time instant makes sense, since the influence of unwanted distortions (e.g. CLC components) between those two sources might vary.

Element-wise PSM basically enhances the shared joint energy bands and diminishes the unshared ones [23]. This technique is advantageous in strengthening shared energy bands, potentially also the BF component. Assume the time–frequency representation of RRI and MSV respectively as $S_{xx}(t_i,f_j)$ and $S_{yy}(t_i,f_j)$, where *i* and *j* represent the time and frequency indices. The spectral multiplication at each time and frequency index can be computed by element-wise multiplication of the amplitude spectrum ($S_{xx}(t_i,f_j) \times S_{yy}(t_i,f_j)$) and is expressed as $\mid S_{xx} \cdot S_{yy}\mid$.

The other fusion method CPSD, quantifies the local synchrony among non-stationary signals and provides information about the shared power among two time series in a given frequency. There are high magnitude values surrounding the correlated components of two time series and low magnitude values nearby uncorrelated components. Let’s assume *x*(*t*) and *y*(*t*) as two zero-mean stationary time series, $S_{xy}(t,f)$ as the CPSD can be defined as (5), where $\mathcal {F}\{.\}$ and *E*[.] indicate Fourier transform and expectation operator [24, 25].

5 $$\begin{aligned} S_{xy}(t,f)=\mathcal {F}_{\tau \rightarrow f}\{E[x(t)y^\star (t-\tau )]\} \end{aligned}$$

Having said that, $S_{xy}(t,f)$ for non-stationary processes can have different definitions and therefore variety of methods exist for $S_{xy}(t,f)$ estimation of non-stationary signals in the literature. In this study we used SPWVD estimator for $S_{xy}(t,f)$, proposed in [25], since it has been used on biosignals.

We quantify the coupling between RRI and MSV signals using squared magnitude of $S_{xy}(t,f)$, assuming that BF is a joint frequency component among those signals. The second last sub-figure of Fig. 2 exemplifies the normalized (column-wise *Z*-score normalization) squared CSPD magnitude for sample sets of RRI and MSV depicted in the first and second sub-figures. Similarly, the normalized spectral multiplication is depicted in the last sub-figure. The coupling of the time series in the spectrums (high energy narrow band) is closely aligned with the reference BF (depicted as dashed lines) in the last two sub-figures. We derive the frequency which owns the maximum power at each time instant as the BF estimation.

## Results

### Data

Since our goal is to evaluate the proposed method in real-life context, data was collected from measurements in different activity protocols. In total, there are 67 subjects (30 female and 37 male) aged from 18 to 60 years who participated in eight different protocol phases, including office, household and sport activities. General physical characteristics of the participants are summarized in Table 1.

In the protocol, 4 min of office work (working with computer) and 4 min of emotional stress (video-induced emotion) comprise the office setting. The household activities consist of floor sweeping, tidying up and table cleaning, each of which lasts for 4 min. The sport setting includes walking, cycling and running that each similarly last for 4 min each. The intensity level of each task is noted in Table 2 as a percentage of maximal HR ($HR_{max}$). A commercially available spirometer, MetaMax 3B (Cortex Biophysik GmbH, Leipzig, Germany) and a Polar H10-based (Polar Electro, Kempele, Finland) prototype which was modified to produce one-lead ECG were used to record the reference respiration and ECG, respectively. The ECG signal voltage resolution was approximately 2.44 μV and it was up-sampled to 1 kHz, and spirometer data was re-sampled to the rate of 1 sample/s.

**Table 1:** General characteristics of participants

Characteristic	Mean	Min	Max
Height (cm)	175	160	195
Weight (kg)	75.4	45.6	122.8
Age (years)	37.9	18	60
BMI (kg/m^2^)	24.51	14.72	35.5

**Table 2:** Table of exercise intensity

Activity protocol	Mean	Min	Max
Office work	45	28	66
Emotional stress	43	26	73
Floor sweeping	52	35	73
Tidying up	54	38	75
Table cleaning	50	34	77
Walking	52	36	74
Cycling	66	48	83
Running	75	48	91

Mean intensity of activity protocols as a percentage of $HR_{max}$

### Evaluation metrics

The constructed series of estimated BF ($\hat{R}=\{\hat{r_1}, \hat{r_2},\hat{r_3},\ldots \}$) possesses the same frequency (number of samples) as the reference BF ($R=\{r_1, r_2, r_3,\ldots \}$) recorded by the spirometer. Thus, we can construct pairs of samples and compute the error between those. Two metrics are computed for the performance evaluation. Percentage error ($\%E$) computed in 6, basically penalizes more for equal error in lower reference BF range.

6 $$\begin{aligned} \%E=\frac{100}{N} \sum _{i=1}^{N} {\left| {\frac{{\widehat{{r_{i} }} - r_{i} }}{{r_{i} }}} \right|} \end{aligned}$$

**Fig. 3:**
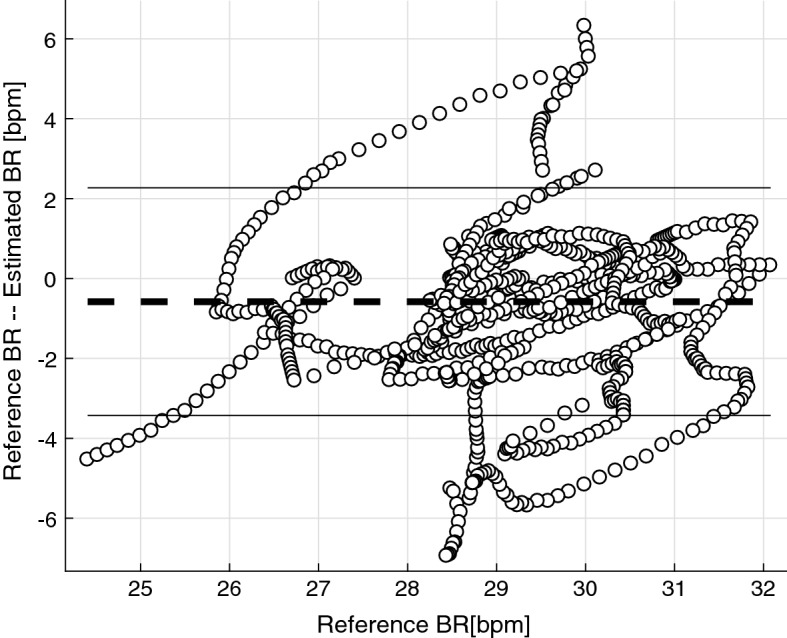
Bland–Altman plot of a sample estimation. The BF estimation performance of a sample time series illustrated as Bland–Altman plot. The thick dashed line indicates the mean deviation value ($\overline{R-\hat{R}}$) and the solid lines represent $\overline{R-\hat{R}} \pm 2\sigma (R)$. In this sample, $\%D^{2\sigma }=14.81$ and $\%E = 5.97$

Additionally, using Bland–Altman plot, the mean deviation is defined as $\overline{R-\hat{R}}$, where the bar denotes the averaging operator. The percentage of pairs of samples differing more than the range of $\overline{R-\hat{R}} \pm 2\sigma (R)$, expressed as $\%D^{2\sigma }$ are reported, where $\sigma (x)$ is the standard deviation of *x*. In Fig. 3 Bland–Altman plot is depicted for a sample set of BF estimation. $\%D^{2\sigma }$ is the percentage of samples beyond the solid line boundaries.

**Table 3:** Table of results

Activity protocol	$\mid S_{xy}\mid ^2$		$\mid S_{xx} \cdot S_{yy}\mid$		$S_{xx}$		$S_{yy}$	
	$\%D^{2\sigma }$	$\%E$	$\%D^{2\sigma }$	$\%E$	$\%D^{2\sigma }$	$\%E$	$\%D^{2\sigma }$	$\%E$
Office work	13.09	7.82	*8.65*	*5.61*	16.48	11.56	14.08	12.21
Emotional stress	13.62	10.98	*9.03*	*8.68*	16.47	11.67	16.70	14.42
Floor sweeping	13.43	11.08	*10.74*	*9.03*	22.17	15.77	18.75	11.57
Tidying up	17.96	13.74	*14.05*	*11.50*	25.37	17.94	21.25	14.18
Table cleaning	16.91	13.01	*9.93*	*9.05*	22.21	15.82	18.51	12.33
Walking	11.31	11.31	*8.51*	*8.74*	17.35	15.99	23.00	16.95
Cycling	8.10	9.44	*5.40*	*6.96*	21.35	16.97	10.10	11.71
Running	15.59	19.17	*12.58*	*16.05*	28.20	24.06	25.16	25.30
Average	13.75	12.06	*9.86*	*9.45*	21.20	16.22	18.44	14.83

The performance of RRI spectral-based ($S_{xx}$) and MSV spectral-based ($S_{yy}$) BF estimation as well as fusion methods, including CPSD-based ($\mid S_{xy}(t,f)\mid ^2$) and spectral multiplication ($\mid S_{xx} \cdot S_{yy}\mid$ ) BF estimation over the protocols. For each method, average $\%D^{2\sigma }$ and $\%E$ is reported and the lowest error in each protocol is written in italics

### Quantitative results

In this subsection, we evaluate the performance of estimated BF by the proposed method. The fusion methods, including squared magnitude of CPSD and the PSM-based BF estimation were compared with the BF estimation from either of RRI or MSV spectrograms [10, 11], using the metrics introduced in the previous subsection. All the software implementation and processing are done in MATLAB R2017a.

Table 3 summarizes the average figures computed for different BF estimation methods. The numbers show that both of the fusion methods outperform the BF estimation from either of the RRI or MSV spectrums exclusively. Among the two fusion methods, the PSM-based BF estimation ($\mid S_{xx} \cdot S_{yy}\mid$) outperforms the other one. Among all the protocol phases, cycling acquires the lowest BF estimation error in the chosen metrics. In contrast, the running and tidying up phases are the most erroneous BF estimation.

## Discussion and conclusion

Computationally efficient methods to boost the performance of BF estimation via ECG surrogate signal processing could enlarge the scope of BF monitoring applications, increase user-acceptance, and provide the users more accurate data. Single-channel ECG-derived BF estimation was investigated in this paper. Unlike many studies in the literature of this discipline, the purpose of our practical approach was to examine the performance of methods in a real-life like context. Thus, our database was comprised accordingly of different real-life settings, including office, households and sport activities.

The existence of breathing component in ECG and feasibility of deriving BF from the RSA and morphological variation of the signal is well-recognized in the literature. Nevertheless, the examination of BF estimation during daily activities measured by a single-channel wearable ECG recorder has not been largely studied. This is most likely due to the challenges involved in such a context such as noteworthy movement artifacts, variable and non-stationary HR, and CLC component’s introduction to ECG.

We proposed to fuse two sources of existing respiratory components in ECG, since those sources might be differently influenced by noise, movements, physiological factors such as age and health, as well as aliasing artifacts. Spectral-domain fusion methods, including CPSD and PSM were applied on RRI and MSV signals, constructed and derived from ECG. Table 3 shows that the performance of fusion methods in all the activity protocols in a daily-life situation is superior to the BF estimation derived from a single source, whether RRI- or MSV-derived BF.

Among the fusion methods, the PSM offers more accurate estimation, compared to the CPSD-based estimation. Both fusion methods are computationally efficient, while relatively PSM technique is slightly more demanding than the CPSD as two separate spectrograms are computed, and then BF is estimated from the multiplied spectrum. In CPSD, the computational cost is reduced by avoiding another transform computation and then the subsequent multiplication of the spectrograms. Quantitatively in a non-optimized implementation, for two time series with 130 s of data, the elapsed time for computation of PSM fusion is 0.61 s versus 0.19 s for CPSD fusion. These numbers were acquired with an Intel(R) Core(TM)i5-3570 processor @ 3.40 GHz and 8 GB of RAM on a 64-bit operating Windows 7.

It should also be noted that whereas the PSM only requires sufficient energies to exist at the same time for a high reading, the CPSD requires also phase difference stability between the two signals [26]. Based on our results provided in Table 3, the superiority of spectral multiplication could hence be explained by phase dispersion between the two derived time series.

## Footnotes

1 In this study, local mean HR is derived from the computed baseline of RRI using a 5-s window moving average smoothing function.
